# Post-Partum Pill

**Published:** 1876-01

**Authors:** 


Post-Partum Pill.—At Bellevue Hospital the following has a popular hospital reputation as a substitute for the regulation dose of castor-oil after parturition:

℞.—Ext. Colocynth Comp., . . . .

Hydrarg. Submuriat., . . . . aa. ʒiij.

Ext. Nucis Vom., . . . .

Pulv. Aloes, . . . . . . .

Pulv. Ipecac, . . . . . . aa. grs. xx.

M. et. div. in pil No. 120. One to four to be taken at a dose. *Medical Record.*

